# Designing and Evaluating a Digital Family Health History Tool for Spanish Speakers

**DOI:** 10.3390/ijerph16244979

**Published:** 2019-12-07

**Authors:** Maria Cerda Diez, Dharma E. Cortés, Michelle Trevino-Talbot, Candice Bangham, Michael R. Winter, Howard Cabral, Tricia Norkunas Cunningham, Diana M. Toledo, Deborah J. Bowen, Michael K. Paasche-Orlow, Timothy Bickmore, Catharine Wang

**Affiliations:** 1 Department of Community Health Sciences, Boston University School of Public Health, Boston, MA 02118, USA; mfcerda@bu.edu (M.C.D.); trevinom@bu.edu (M.T.-T.); cbangham@bu.edu (C.B.); tln@bu.edu (T.N.C.); 2 Health Equity Research Lab, Cambridge Health Alliance, Cambridge, MA 02141, USA; dharma_cortes@hms.harvard.edu; 3 Department of Psychiatry, Harvard Medical School, Cambridge, MA 02139, USA; 4 Biostatistics and Epidemiology Data Analytics Center (BEDAC), Boston University School of Public Health, Boston, MA 02118, USA; mwinter@bu.edu; 5 Department of Biostatistics, Boston University School of Public Health, Boston, MA 02118, USA; hjcab@bu.edu; 6 Department of Pathology and Laboratory Medicine, Dartmouth-Hitchcock Medical Center, Lebanon, NH 03756, USA; Diana.M.Toledo@hitchcock.org; 7 Department of Bioethics and Humanities, School of Public Health, University of Washington, Seattle, WA 98195, USA; dbowen@uw.edu; 8 Department of Medicine, Boston University School of Medicine, Boston, MA 02218, USA; mpo@bu.edu; 9 College of Computer and Information Science, Northeastern University, Boston, MA 02115, USA; bickmore@ccs.neu.edu

**Keywords:** family health history, e-health, Spanish language, health literacy, health disparities, conversational agent, genetic communication, ethnic/racial minorities, evaluation of genomic tools for public health, public health genetics

## Abstract

Digital family health history tools have been developed but few have been tested with non-English speakers and evaluated for acceptability and usability. This study describes the cultural and linguistic adaptation and evaluation of a family health history tool (VICKY: VIrtual Counselor for Knowing Your Family History) for Spanish speakers. In-depth interviews were conducted with 56 Spanish-speaking participants; a subset of 30 also participated in a qualitative component to evaluate the acceptability and usability of Spanish VICKY. Overall, agreement in family history assessment was moderate between VICKY and a genetic counselor (weighted kappa range: 0.4695 for stroke—0.6615 for heart disease), although this varied across disease subtypes. Participants felt comfortable using VICKY and noted that VICKY was very likeable and possessed human-like characteristics. They reported that VICKY was very easy to navigate, felt that the instructions were very clear, and thought that the time it took to use the tool was just right. Spanish VICKY may be useful as a tool to collect family health history and was viewed as acceptable and usable. The study results shed light on some cultural differences that may influence interactions with family history tools and inform future research aimed at designing and testing culturally and linguistically diverse digital systems.

## 1. Introduction

Family health history is one of the most important risk factors for many chronic conditions, including cardiovascular disease, diabetes, and cancer [1,2,3]. There is a substantial burden of family-history based risk in primary care populations, which has important implications for disease screening and management. For example, the Family Healthware^TM^ Impact Trial reported that 82% of participants who completed the risk assessment tool had an elevated familial risk for one of the six diseases assessed (coronary heart disease, stroke, diabetes, breast/colon/ovarian cancers) [4]. Despite this burden, the collection of family history information by patients and the integration of family history assessment into clinical practice has been surprisingly poor in frequency and quality [5]. Several barriers preclude the systematic documentation of family history in clinical settings, including lack of time, lack of compensation for the effort, clinicians’ lack of knowledge and skills, and other logical barriers, such as lack of standardization in family history collection methods [5,6,7,8].

Due to the importance of family history assessment and its lack of systematic documentation, several efforts have been undertaken to develop digital family history tools to improve the documentation and use of family history [9]. Although these tools have mostly been developed as public or patient-facing tools, there is strong evidence to suggest that they may not be accessible to a large portion of the general population with limited health literacy or computer skills, due to high reading grade demands and navigational challenges [10,11,12,13,14]. 

In addition to these challenges, the number of family history tools in languages other than English is limited and the lack of availability of digital tools for non-English language speakers serves as an additional impediment to their accessibility and may inadvertently widen inequities in health [15,16]. To date, very few family history tools are available in Spanish and those that are available have not been evaluated among Spanish speakers [17].

We have previously developed and pilot tested a prototype virtual counselor (conversational agent) to collect family history information (VICKY: VIrtual Counselor for Knowing Your Family History). VICKY 1.0 was shown to have good acceptability and feasibility among English speaking patients at a safety-net hospital [18]. Others have similarly shown that conversational systems are more usable compared to traditional interfaces for compiling family health histories [19]. Currently, VICKY 2.0 is undergoing a randomized controlled trial to determine its accuracy in collecting health history information compared to another publicly available digital tool (My Family Health Portrait, https://phgkb.cdc.gov/FHH). 

As part of an expanded effort to develop tools to reach non-English speaking populations, we undertook the development of VICKY in Spanish. As the largest minority in the US, representing 18.3% of the US population in 2018 (59.9 million) [20], Latinos experience health disparities that have been associated with limited access to health services in general and preventive care services in particular [21], as well as linguistic barriers, including the lack of translators and linguistically appropriate materials and tools that may facilitate interactions with healthcare providers [22]. The most recent reports show that Latinos are at high risk for non-communicable diseases, such as heart disease, cancer, high blood pressure, diabetes mellitus, chronic respiratory disease, all of which account for 58% of all deaths among Latinos [23]. Given that approximately 41 million individuals in the United States (about 14% of the total population) speak Spanish at home [24], the development and evaluation of family history tools in Spanish was critical. 

The purpose of this paper is threefold: (1) to describe the adaptation of VICKY for Spanish speakers, (2) to evaluate the agreement of family histories collected by Spanish VICKY compared to those collected by a genetic counselor, and (3) to describe the acceptability and usability of the tool.

## 2. Materials and Methods 

### 2.1. VICKY Program—System Architecture

**Figure 1:**
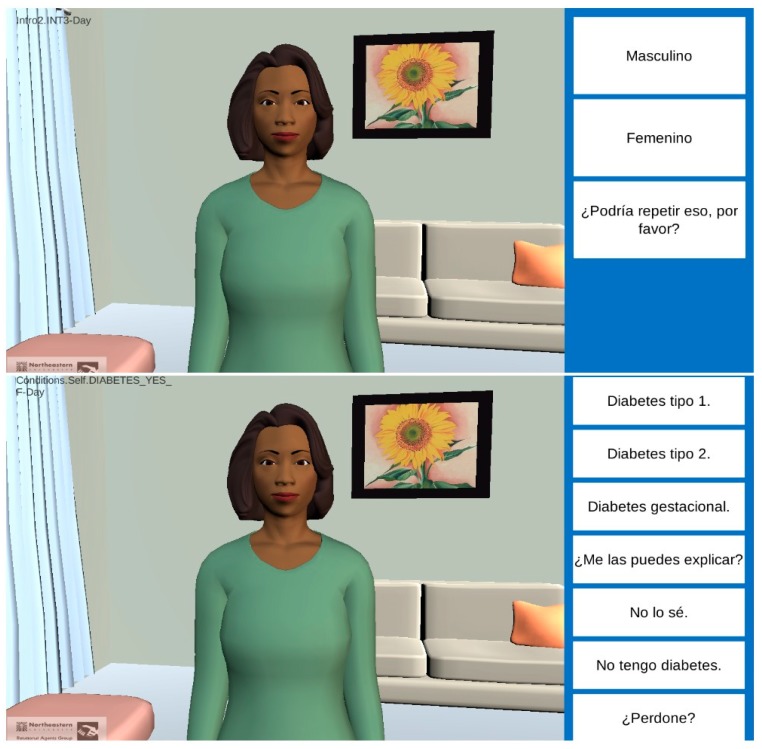
Screenshots of Spanish VIrtual Counselor for Knowing Your Family History (VICKY).

VICKY (VIrtual Counselor for Knowing Your Family History) is a web-based conversational agent that uses a browser-based animation system for the animated character and user interface, together with a server-based dialogue engine, database and speech synthesizer (see Figure 1). The dialogue is designed using hierarchical transition networks to allow dialogue to be modeled in layers of abstraction [25], together with template-based text generation [26] to allow for the tailoring of individual utterances for each user. Agent nonverbal conversational behavior, such as hand gestures, facial displays, and gaze behavior, is generated primarily using the BEAT (Behavior Expression Animation Toolkit) automated text-to-embodied-speech translation system [27]. User input is obtained primarily through a multiple-choice menu of response options updated at each turn of the conversation, allowing all dialogue to be thoroughly validated (unconstrained natural language input can pose a safety risk for patient-facing medical counseling systems [28]). 

### 2.2. Adaptation and Refinement for Spanish Speakers

Following the finalization of the English version of VICKY 2.0, the tool was translated into Spanish by an experienced doctoral level translator with expertise on health literacy issues, cultural adaptations and health communication. The goal of the translation was to produce a text with three levels of equivalence: content (i.e., questions asked are relevant to Spanish speakers), semantic (i.e., intended meaning of questions asked is the same in both cultures), and technical (i.e., data collection method is appropriate across cultures) [29,30,31,32]. To achieve this goal, the translation process entailed two steps: a forward translation by one single senior translator and a translation review by two independent bilingual researchers, a method that has been used to translate outcome measures [31]. This team consisted of individuals originating from Puerto Rico, Venezuela and Chile (with family originating from Mexico).

For the VICKY tool, the English content includes no untranslatable idiomatic expressions nor culturally bound terms or regionalisms that would require complex linguistic adaptations to render the same meaning. However, during the translation process, careful attention was given when translating certain words such as “child and children” from English into Spanish. The literal (and correct) translation of child/children is “*niño/niños*.” However, the use of *niño/niños* is not specific enough as to restrict its use to children related by blood. Since VICKY’s goal was to collect family history of genetically related individuals and adopted children, the words “child” and “children” were translated as *hijo/hija* (son/daughter) and *hijos/hijas* (sons/daughters) to communicate in unequivocal fashion that the questions pertain to biological children (i.e., when VICKY is not asking about adopted children). Linguistically, this level of wording specificity was important for a tool designed to generate information for genetic counselors and other health care providers, to make sure that non-biological children raised as family members, commonly known as “*hijos de crianza*” among Latinos, are not reported as biological children. 

Throughout the translation process, other language-related decisions were made based on Spanish language conventions that convey social norms and expectations based on age differences, formality of a relationship, and social position. For example, the manner in which VICKY addresses users was modified due to the computer agent’s appearance as a young adult female. Since it was likely that most users would be older than VICKY appears, the Spanish translation incorporated the formal version of the second person pronoun (you) “*usted*” instead of “*tú*” (informal version) when VICKY addresses users. “*Usted*” conveys the type of formality most Spanish speakers expect when interacting with a new acquaintance and older people. Accordingly, in light of VICKY’s younger appearance, the written responses for participants to reply to the system uses “*tú*”. 

VICKY’s final Spanish version was reviewed by two independent bilingual researchers for accuracy. Recommendations to modify the translated text (i.e., mostly verb conjugations) were reviewed and finalized together as a team, with the goal of producing an international or neutral Spanish version of VICKY. Translation efforts as a team allowed for fluid and more inclusive and homogeneous communication among Spanish speakers from different countries [33,34]. The team also provided needed input to select the text-to-speech voice for VICKY from two available CereProc [35] voice options (i.e., Castilian and Mexican); and selected the Mexican option after deeming it to represent more neutral sounding Spanish that would be acceptable to a broad array of Spanish speakers. More specifically, this option was selected because the voice resembled phonetic sounds and minimized phonetic differences across Spanish accents, similar to what is achieved by panhispanic media outlets [36].

### 2.3. Study Procedures

The present study was a supplemental study conducted alongside a parent randomized controlled trial to assess the validity, acceptability and usability of Spanish VICKY. All study participants gave their informed consent for inclusion before they participated in the study. The study was conducted in accordance with the Declaration of Helsinki and the protocol was approved by the Institutional Review Board of Boston University Medical Center (H-32767).

Study participants were recruited via one of three methods. The first method focused on identifying Spanish speaking patients though the patient database at a safety net hospital. Specifically, following approval from physicians, recruitment letters describing the study were mailed to patients who could opt out or contact the research team directly if interested in participating. A second recruitment method entailed distributing flyers to Spanish-speaking primary care physicians at the hospital to post or distribute in clinics. Flyers were also distributed at community centers, public libraries, grocery and convenience stores, among others. Finally, snowball recruitment was utilized, wherein participants were encouraged to inform others about the study.

Study eligibility criteria included 21 years of age or older, preference for speaking Spanish with a physician, not having a vision impairment preventing the use of a computer, no significant cognitive impairments, and no previous participation in a study using VICKY. Prior experience with computers was not used for screening eligibility as the purpose of the study was to determine if the tool could be used by individuals who ranged in their proficiencies with computers. Efforts were made during the screening process to recruit participants from a diverse range of Spanish-speaking countries. Study eligibility was assessed by research assistants over the telephone prior to study consent. 

Study participation involved a single in-person interview lasting approximately 3 h. During the visit, participants consented and asked a series of baseline questions. Next, participants completed their family health history using Spanish VICKY. Time spent on Spanish VICKY was recorded by the VICKY system. Upon completion of the tool, a post-tool survey was administered, followed by a conversation with a bilingual genetic counselor via videoconference, who collected a family health history following a structured interview protocol (see Supplemental Material S3) for the purpose of ascertaining the accuracy of Spanish VICKY. After the conversation, a subset of the participants were invited to participate in a qualitative component of the interview to evaluate the acceptability and usability of the tool. Survey interview items (e.g., demographics, health literacy) and closed-ended questions asked during the qualitative interview were recorded in REDCap (Research Electronic Data Capture, https://www.project-redcap.org/).

### 2.4. Study Measures 

Participant demographics, including gender, age, education, and income, were collected using standard measures. Health literacy was assessed using the Newest Vital Signs, which has been validated in Spanish for identifying people with limited health literacy skills [37]. Computer and internet literacy and access was assessed using a 6-item tool adapted from prior work [38].

The qualitative component of the interview consisted of open and closed-ended questions and focused on ascertaining issues pertaining to content and navigation of Spanish VICKY. The open-ended questions included in the interview focused on the acceptability and usability of the tool (e.g., What did you like most/least about VICKY? How easy or difficult was it to use VICKY? What was difficult or confusing about VICKY? Would you prefer VICKY in Spanish or English?). The closed-ended questions were investigator-adapted items utilized regularly by the research team to assess the acceptability of relational agents (e.g., How much did you like VICKY? How comfortable were you talking with VICKY about your family? How similar is VICKY to you? How much do you feel you trust VICKY? How satisfied are you with VICKY?). These questions were then followed with open-ended prompts to assess factors that contributed to the ratings provided by participants. All qualitative interviews were audio recorded, transcribed verbatim, and analyzed in Spanish to better ensure that the meaning as conveyed by participants was maintained during the coding process. Quotes were selected in Spanish to illustrate themes identified and were subsequently translated into English with the goal of achieving faithfulness (i.e., rendering the meaning of the source text) and transparency (i.e., conforming to the target language’s grammar and syntax) by the senior bilingual translator on the project.

### 2.5. Data Analysis

#### 2.5.1. Demographics and Acceptability Rating Scales

Analyses of demographics and rating scales were conducted to generate descriptive statistics including counts and percentages for categorical variables, as well as means, standard deviations, and ranges of responses where appropriate for continuous variables. 

#### 2.5.2. Agreement between Pedigrees

For participants with pedigrees from both VICKY and the genetic counselor, agreement between pedigrees of family members identified was assessed as follows. First, the number of VICKY-identified relatives was divided by the number of relatives identified by the genetic counselor. Agreement rates were derived for first-degree relatives, second-degree relatives, and a combined total of first- and second-degree relatives.

Agreement between pedigrees for health conditions was examined by first categorizing the number of first-degree relatives in each pedigree with a given condition as 0, 1, 2, and 3 or more. Then, four by four cross-tabulation tables were constructed and weighted kappas and corresponding 95% confidence intervals were calculated for each condition [39]. Agreement was described by calculating, for each condition, how many pedigrees had perfect agreement, how many agreed within one case, how many agreed within two cases, and how many agreed within three or more cases.

All quantitative statistical analyses were conducted using SAS/STAT software, version 9.4 of the SAS System for Microsoft Windows (SAS Institute, Cary, NC, USA). 

#### 2.5.3. Qualitative Data 

Qualitative data collected through the interviews were analyzed in Spanish following a three-step thematic data analysis conducted by two independent coders and a third senior-level qualitative data researcher, all of whom are bilingual (Spanish/English) speakers. The first step involved independent open coding of 12 transcripts performed by two coders. The goal of this step was to identify major thematic areas and to develop the codebook containing the codes and sub-codes that would be used to analyze and interpret the collected data. The narrow scope of the interview guide (i.e., assessing tool acceptability and usability) resulted in a thematic codebook that closely matches the interview guide. Nonetheless, the goal of the thematic analysis was to extract passages that captured participants’ perceptions of and experiences using VICKY. Once the final codebook was established and the two coders reached agreement (k = 0.81), the remaining interviews were coded by a single coder (second step). All coding was conducted using NVivo version 12 (QSR International, Melbourne, Australia). The third step involved identifying relationships and patterns among the open codes by a senior qualitative data researcher who reviewed all coded interviews in order to qualitatively determine whether users of Spanish VICKY deemed the tool acceptable and useable. 

## 3. Results

### 3.1. Tool Refinement and Initial Comparisons with English Language Tool

Due to differences in grammar, sentence structure, and word length between English and Spanish, the translation of VICKY into Spanish led to text expansion compared to the source language and resulted in a longer tool. The resulting Spanish version added approximately 10–20 min in length of time to complete the *same* family history in English versus Spanish, when tested by the research team. 

In addition, observations by team members noted much larger family sizes among the Spanish-speaking participants, which then resulted in more time spent entering family histories within Spanish VICKY (i.e., approximately 60 min on average vs. 50 min for patients using English VICKY).

### 3.2. Interviews 

#### 3.2.1. Demographics

A total of 56 Spanish-speaking participants took part in the study, of which 30 also completed the qualitative component. Table 1 presents the demographics of study participants. Overall, 66% were female, 53% were 45 years and older, 62% had a high school degree or less, and 63% had a household income of $35K or less. Approximately one-third of participants had limited health literacy and over half reported limited computer experience. Half of participants (28/56) were recruited from the community-based efforts (flyers distributed at community centers, public libraries, grocery and convenience stores); and half were recruited from mailed recruitment letters to patients. 

#### 3.2.2. Agreement between Spanish VICKY and Genetic Counselor

One participant was unable to use VICKY and therefore, no VICKY pedigree was generated. This participant was not part of the subset of 30 individuals who completed the qualitative component of the interview. 

##### Agreement—Family Members Identified

In comparison with a genetic counselor, VICKY identified 460/456 (100%) first-degree relatives and 1316/1264 (104%) second-degree relatives, for a total of 1776/1720 (103%) relatives combined. 

##### Agreement—Conditions Identified

Table 2 presents the results on the agreement between Spanish VICKY and genetic counselor for six disease conditions. Overall, agreement was moderate to good, with weighted kappas ranging from 0.4695 (95% CI: 0.1677–0.7712) for stroke to 0.6615 (95% CI: 0.5029–0.8202) for heart disease (framed as heart problems for a lower literate patient population). Additional analyses examining agreement by diabetes and cancer subtypes revealed wide variation depending on the subtype in question (see Supplemental Table S1). For example, agreement was perfect for colon cancer (weighted kappa = 1.0000), yet poor for prostate cancer (weight kappa = 0.1996, CI: −0.0907–0.4899). 

#### 3.2.3. Acceptability and Usability of Spanish VICKY

##### Likeability

Participants were asked to rate how much they liked VICKY using a scale of 1–10 (1 = not at all and 10 = very much) and rated likeability at 9.1 (Standard Deviation SD = 1.1; Range = 7–10). When asked about the features that they liked, participants mentioned several areas: VICKY’s use of plain language, the questions she asked were “brief and clear”, the multiple-choice response format, user friendliness, and not feeling pressed by time. Examples to illustrate general areas of likability are presented in Table 3.

Eleven participants (37%) noted an almost human-like connection. One participant’s description of how it felt to interact with VICKY captured two key elements that seemed to contribute to the high levels of likeability participants reported.

*In its virtual reality [VICKY] is someone who has many human characteristics like us, and the way [she] communicates is very simple. (Female, age 35–44, possible limited literacy, uses computer regularly).*

Another reason that led participants to describe the connection with VICKY was based on the fact that VICKY expressed emotions such as sympathy when participants reported information about a deceased relative. In fact, about half of the participants reported that her expressions of sympathy helped them alleviate the sadness and nostalgia they experienced when having to report that a relative was deceased.

*I liked that VICKY had the sensitivity to also understand that, that when she asked a question that could be somewhat uncomfortable, like the death of a relative, she was sensitive in that aspect. So it was an interesting experience that an impersonal electronic system can be almost real. (Male, age 65 +, limited literacy, computer expert)*

##### Comfort Level and Trust

When participants were asked how comfortable they felt talking to VICKY about their family history (from 1 to 10: 1 = not at all comfortable, 10 = very comfortable) they rated their comfort level very highly (Mean = 9.5; SD = 0.8; Range = 7–10). They also reported high levels of trust (Mean = 9.6; SD = 0.8; Range = 7–10). VICKY was described as non-threatening and trustworthy, especially due to her virtual nature.

*[One] does not have to be scared or anything like that … there’s nobody looking at you or anything like that, it’s a computer person. It’s like talking to nobody, but they’re listening to you … I wanted to talk without feeling like someone is watching me … I felt comfortable. (Male, age 25–34, limited literacy, uses computer regularly)*

*I liked it because it opened my mind … because it was difficult for me to start communicating with my doctor, right? And this I liked, I do not know, it made me feel open even about myself, to talk about my health. (Female, age 45–54, limited literacy, tried computer a few times)*

One other participant felt that using VICKY safeguards confidentiality of shared information.

*[I trust] a lot, it seems to me that it is something that will not come out of there and that is something confidential, and that remains on the computer and nobody else knows it. Nobody else is going to say it. (Female, age 35–44, possible limited literacy, uses computer regularly)*

##### Preference for Gender and Language

Participants were asked if they had preferences regarding VICKY’s language and gender. All participants reported that, given the option between Spanish and English, they would prefer to speak in their native language, Spanish, except for one participant that said there were terminologies that they understood better in English. Participants generally reported that they had no preference as to VICKY’s gender (*n* = 23, 77%).

##### Navigation

All participants reported that VICKY was very easy to navigate. Additionally, participants were highly satisfied with VICKY’s clarity of instructions (Mean 9.8; SD = 0.6; Range 7–10). All the participants reported that the language was simple and clear and that the sequence was well organized. However, a recurring issue that eight participants (27%) discussed was not being able to figure out how to fix mistakes when entering information.

*What is difficult about VICKY was that I noticed that in some instances I made a mistake and could not go back, that’s what made it a bit difficult, and I could not correct what I had entered. (Female, age 35–44, possible limited literacy, tried a computer a few times)*

##### Completion Time

The majority of participants (27/30) reported that the length of time it took to complete the tool was “just right”. Notably, over one third of participants with high levels of computer skills reported a delayed response (time lag) on VICKY’s part when answering questions and no options to increase the speed at which VICKY moves between screens or to skip sections.

*A little slow. I feel that it took much longer than it should. (Female, age 35–44, possible limited literacy, uses computer regularly)*

##### Interface Improvements and Customization

Although most participants were satisfied with the VICKY interface (i.e., touchscreen, multiple-choice menu of “response” options), seven participants indicated that they would have liked to use voice commands to respond to VICKY.

*Without touching the screen but from voice to voice, like that. (Female, age 45–54, adequate literacy, uses computer regularly)*

One third of participants mentioned that they felt that VICKY could share more information about disease management as well as resources to address health concerns.

*I would like you to give me information about what I can do about a problem within my family … (Male, age 25–34, limited literacy, tried computer a few times)*

Participants also suggested ways to customize VICKY’s appearance to their liking. For example, more than half of participants (53%) suggested ways in which VICKY could be made more “real” by improving nonverbal communication such as increased movement of her mouth and hands when she talks, as well as smiling.

*Maybe because Latinos talk with their hands, maybe yes, if they use their hands a little when they speak, because Latinos … express themselves a lot when they talk with their hands, I do not know, move them. (Female, age 35–44, possible limited literacy, tried a computer a few times)*

*Yes, very serious. It’s better if she puts on a smile or something there. (Male, age 25–34, limited literacy, uses computer regularly)*

Others recommended adding features that would allow them to alter VICKY’s hair color, skin color, and accent in order to make her more similar to them. Nonetheless, participant ratings of the extent to which VICKY was similar to them was also high (Mean = 8.2; SD = 2.4; Range = 1–10).

## 4. Discussion

Digital solutions for documenting family health history have the potential to identify an important risk factor for chronic disease prevention and management, yet are often not accessible to underrepresented patient populations due to inherent literacy and language-related barriers. Prior work has demonstrated that culturally informed Spanish-speaking conversational agents are an acceptable approach for providing health education and encouraging changes in health behavior among underserved communities [40,41]. The present study builds on this work by providing evidence that Spanish-speaking agents are feasible as a tool for collecting complex family health history information, which is important in the effective prevention and management of health.

This study set out to describe the cultural and linguistic adaptation of VICKY for Spanish speakers and evaluate its acceptability, usability, and agreement with a genetic counselor. Overall, the family health histories ascertained by VICKY were in moderate agreement with histories obtained by the genetic counselor, often with disagreement occurring because of a single disease case among first degree relatives that differed between the pedigrees collected. Notably, the pattern was not consistent with regards to which pedigree was missing the case reported, suggesting that disagreement may reflect situations where conditions were missed by either VICKY or the genetic counselor. Certain variations did, however, suggest areas where Spanish VICKY warrants improvement; namely, the ascertainment of Type 2 diabetes and prostate cancer. For example, in the case of Type 2 diabetes, it appeared that many patients reported Type 1 diabetes instead of Type 2 to VICKY, suggesting that there is confusion about the distinction between the two that a genetic counselor might ask follow-up questions to clarify. Future digital tools require better clarification for patients about diabetes. 

There were several aspects of Spanish VICKY that were important to evaluate, including whether the increased length of time it took to enter family history would be too burdensome. The results of this study demonstrate that in spite of the greater length of time it took to use Spanish VICKY, participants were overwhelmingly positive in their responses, felt that the length of time was appropriate, and preferred a Spanish language version of the tool (among those who could also speak English). Future efforts to disseminate the tool for widespread use may need to revisit the issue of completion time and consider programming strategies (e.g., allowing for breaks or completion of family histories across different time points) to mitigate any possible challenges with time burden.

An important strength of this study was that study participants varied in nationalities and were purposively recruited from different Spanish-speaking countries. Even with this variation, all the participants felt that VICKY was culturally and linguistically appropriate. Some participants suggested improvements to the tool, including the ability to customize VICKY’s physical appearance (hair and skin color) and accent to make her more representative of their country. Others suggested making VICKY gesture and speak in a manner that was culturally similar. These considerations, however, did not detract from participants’ favorable impression of VICKY overall. Nonetheless, the ability to customize these aspects in future conversational agent platforms may further improve user’s perceptions of cultural sensitivity, level of connection with the program, and the ultimate impact of the tool [42]. Moreover, because Spanish VICKY was developed and tested in the United States, further testing is warranted to confirm the appropriateness of VICKY in cultural and linguistic contexts outside of the US. 

The use of Spanish-language conversational agents can overcome many of the aforementioned literacy-related barriers [15,43] and increase the access and use of digital family history tools among traditionally marginalized groups. Although our Spanish-speaking participants had different backgrounds and varied in education and health literacy, almost all of them (55/56, 98%) completed VICKY successfully. Many of the participants expressed that the organization and language used was clear and easy to follow because it went “step by step”. Moreover, having written choice options on the screen, the chance to have VICKY repeat what was said, and the opportunity to hear explanations of health conditions all contributed to the positive navigation experience for participants. There was, however, a single participant who could not use the Spanish VICKY program. Notably, this individual had limited health literacy and reported an education level of 9–12th grade with no high school diploma or equivalent. This participant did report using a computer regularly, however, this reflected the use of a smartphone device as compared to a computer (touchscreen) platform. As such, although the VICKY program appeared to overcome many barriers to accessing digital family history tools, challenges nonetheless remain for some potential users. Future efforts may benefit from exploring mobile platforms to document family history information, which may be more familiar and accessible to individuals with less education and limited health literacy [44,45,46,47]. 

Some navigation challenges were noted that are important to address in future efforts. In particular, participants expressed challenges in fixing mistakes they had made when entering their family history. In the conversational format of the VICKY script, there was not an option for a “back button” per se, allowing participants to immediately correct their error. Although VICKY states early in the program that any mistakes can be corrected later, and circles back to ask about any changes or edits that need to be made to the family tree that is generated, this format to correct mistakes seemingly caused some distress for some participants and likely contributed to errors in data entry. 

VICKY was perceived as non-judgmental and humanlike (including expressions of empathy), all of which facilitated acceptance and engagement with the tool. Moreover, the use of a conversational agent like VICKY generated interest in learning more information about health conditions and may facilitate the longer-term use of digital solutions among underserved groups to promote better health and disease management, particularly among those who have limited literacy. Many participants indicated they did not know about family history trees (pedigrees) prior to using VICKY and were motivated to talk to their family members and doctors about their family health history. Communication outcomes following the use of a tool like VICKY are being examined in the parent trial. VICKY, as with other family history tools, can serve as a trigger to get individuals to think about their health and motivate them to engage in conversations with family members and health care providers to learn more information [48]. Future research efforts should focus on identifying where to disseminate and how best to implement family health history tools like VICKY to ensure broader reach and impact.

There were several limitations to the present study that should be noted. First, study research assistants (RAs) were physically present when participants were using VICKY. Although RAs could not provide any assistance on how to use the tool, there were instances when participants asked for help (e.g., how to start VICKY, what to do when they made a mistake). Although RAs were instructed not to intervene if participants were “stuck” (since this was part of usability), they did assist on getting participants started if necessary in order to be able to interview on the acceptability of the tool. In addition, tool usability was not tracked with usability software but from observational notes made by the RAs. As such, there may have been usability issues that were missed by the RAs and not reported by participants during the qualitative interviews. 

Second, the subset of participants who took part in the qualitative interviews was mostly recruited from the community via flyers and word of mouth (22/30). Our team noted some differences between the community-recruited participants and the patient participants recruited from the hospital. In particular, community participants were younger and felt more skilled at using the Internet compared to patient participants. They were also more likely to be from Central American countries (e.g., El Salvador, Guatemala, Honduras) as opposed to Caribbean countries/US Territories (e.g., Puerto Rico, Dominican Republic). In addition, community participants tended to report smaller family histories, which may have affected tool completion time and overall experience with VICKY. In spite of these differences, no significant differences were noted between recruitment populations on quantitative ratings pertaining to VICKY acceptability and usability. Moreover, we were able to successfully recruit participants for the qualitative interviews from a range of Spanish-speaking countries, with variation across demographics including education, health literacy and computer skills (see Supplemental Table S2). Nonetheless, the differences noted between the community-recruited population and safety-net hospital patient populations suggest that some caution is warranted when generalizing qualitative study findings. 

Third, the sample size for this study was relatively modest and not large enough to compare across Spanish-speaking countries. As such, although we were able to recruit participants representing eight different Spanish speaking countries/US Territories, the results from this study may not be generalizable to the entire range of Spanish speaking countries. Further research may be needed to ensure the appropriateness of the tool for individuals from the other countries not represented in the current study.

Finally, our evaluation of VICKY did not use traditional validation metrics such as sensitivity and specificity, but rather focused on ascertaining the agreement between the family histories collected within Spanish VICKY and those collected by a genetic counselor “gold standard”. Prior research has demonstrated that family health history tools can often collect more information than genetic counselors [49], particularly in the context of stigmatized conditions [18]. Application of these traditional validation metrics assumes that any disagreement is due to errors in the tool pedigree as opposed to errors in the documentation of family history by a human. Unlike in the context of diagnostic screening and testing where the true state of disease may be determined, this approach is less appropriate when a comparator gold standard is potentially inaccurate, as it would make any tool appear less accurate that it is. As part of the parent trial, our team is auditing a subset (20%) of interviews conducted by the genetic counselor in efforts to understand the sources of error and discrepancy between the two documentation approaches, and noting that there is variation in the sources of error across health conditions (Wang et al, manuscript in preparation). As such, agreement in the present study does not fully represent the true accuracy of participants’ family health history.

## 5. Conclusions

In summary, the Spanish language version of VICKY may be a useful tool to collect family health history and is acceptable and usable among Spanish speaking participants. The study results also shed light on some cultural differences that may influence interactions with family history tools and inform future research aimed at designing and testing different language versions of these tools. Ultimately, improving the accessibility of family history tools will help to mitigate a digital divide and overcome an important barrier that often serves to magnify inequities in health. 

## Figures and Tables

**Table 1:** Participant demographics (*N* = 56).

Gender	N (%)	Country/US Territory of Origin	N (%)
Male	19 (34%)	Puerto Rico	20 (35.7%)
Female	37 (66%)	Dominican Republic	14 (25%)
		El Salvador	7 (12.5%)
**Age**		Guatemala	2 (3.6%)
21–24	3 (5.4%)	Honduras	2 (3.6%)
25–34	12 (21.4%)	Ecuador	1 (1.8%)
35–44	11 (19.6%)	Brazil	1 (1.8%)
45–54	10 (17.9%)	Colombia	1 (1.8%)
55–64	13 (23.2%)	Mexico	8 (14.3%)
65+	7 (12.5%)		
**Education**		**Health literacy**	
<9th grade	10 (17.9%)	High likelihood of limited literacy	12 (21.4%)
9–12th grade, no diploma	10 (17.9%)	Possibility of limited literacy	7 (12.5%)
High school diploma or equivalent	15 (26.8%)	Almost always adequate literacy	37 (66.1%)
Some college, no degree	4 (7.1%)		
Associate degree	2 (3.6%)		
Bachelor’s degree	9 (16.1%)		
Graduate degree	3 (5.5%)		
Post graduate degree (doctorate)	3 (5.5%)		
**Income**		**Computer experience**	
$25,000 or less	27 (48.2%)	Never used one	13 (23.2%)
$25,001–$35,000	7 (12.5%)	Tried one a few times	18 (32.1%)
$35,001–$50,000	2 (3.6%)	Use one regularly	20 (35.7%)
$50,001–$75,000	3 (5.4%)	I’m an expert	5 (8.9%)
No answer	17 (30.4%)		

**Table 2:** Agreement between Spanish VICKY versus Genetic Counselor for health conditions (*N* = 55 pedigrees).

Conditions Identified among First Degree Relatives (Number of Cases per Pedigree)	VICKY (Number of Pedigrees)	Genetic Counselor (Number of Pedigrees)	Distribution of Agreement		Weighted Kappa (95% CI)
Heart Problem (including heart attack)					
0	35	33	Perfect agreement	41	0.6615 (0.5029, 0.8202)
1	10	16	Within 1 case	12	
2	7	1	Within 2 cases	2	
3+	3	5	Within 3+ cases	0	
Stroke					
0	46	49	Perfect agreement	47	0.4695 (0.1677, 0.7712)
1	7	5	Within 1 case	7	
2	2	0	Within 2 cases	1	
3+	0	1	Within 3+ cases	0	
Diabetes ^1^					
0	32	23	Perfect agreement	34	0.5911 (0.4295, 0.7527)
1	13	18	Within 1 case	18	
2	5	6	Within 2 cases	3	
3+	5	8	Within 3+ cases	0	
Cancer ^2^					
0	49	45	Perfect agreement	48	0.6590 (0.4665, 0.8515)
1	4	6	Within 1 case	7	
2	1	4	Within 2 cases	0	
3+	1	0	Within 3+ cases	0	
High Blood Pressure					
0	20	20	Perfect agreement	35	0.6556 (0.5156, 0.7956)
1	17	13	Within 1 case	17	
2	9	12	Within 2 cases	3	
3+	9	10	Within 3+ cases	0	

^1^ Includes Type 1 and Type 2 diabetes. ^2^ Includes breast, ovarian, colon, prostate, skin and lung. No cases of breast or ovarian cancer were reported.

**Table 3:** Quotes illustrating likeability.

Likability Area	Illustrative Quote
Use of plain language	*the clear way [she] asks the questions: very simple and very precise*
Multiple choice response format	*[she] would ask the question and would provide options clearly*
User friendly	*everything was step by step*
Not feeling pressed for time	*VICKY has the time to listen to you and you can take your time to answer the questions, things you do not have with the doctor.*
Human-like connection	*What I liked most about VICKY was that she looked more or less like a person … [VICKY] gave me that feeling that I was talking to a live person.*

## Supplementary Materials

The following are available online at https://www.mdpi.com/1660-4601/16/24/4979/s1, Table S1. Agreement by diabetes and cancer type; Table S2. Participant demographics by full sample versus subset who did or did not completed qualitative interview; Supplemental Material S3: Genetic Counselor Script.
