# Supplementary material for: Designing and Evaluating a Digital Family Health History Tool for Spanish Speakers
Source: Int J Environ Res Public Health. 2019 Dec 7;16(24):4979. doi: 10.3390/ijerph16244979 (PMC6950582; doi:10.3390/ijerph16244979)
Supplement: Supplementary file 1 [file ijerph-16-04979-s001.pdf]

**Supplemental Table 1: Agreement by diabetes and cancer type**

| Conditions identified among first degree relatives (# cases per pedigree) | VICKY (# of pedigrees) | Genetic Counselor (# of pedigrees) | Distribution of agreement | | Weighted Kappa (95% CI) |
|---|---|---|---|---|---|
| Type 1 Diabetes | | | | | |
| 0 | 49 | 54 | Perfect agreement | 48 | -0.0280 (-0.0702, 0.0141) |
| 1 | 4 | 0 | Within 1 case | 4 | |
| 2 | 1 | 0 | Within 2 cases | 1 | |
| 3+ | 1 | 1 | Within 3+ cases | 2 | |
| Type 2 Diabetes | | | | | |
| 0 | 36 | 24 | Perfect agreement | 31 | 0.5205 (0.3433, 0.6977) |
| 1 | 10 | 18 | Within 1 case | 21 | |
| 2 | 5 | 6 | Within 2 cases | 3 | |
| 3+ | 4 | 7 | Within 3+ cases | 0 | |
| Colon Cancer | | | | | |
| 0 | 54 | 54 | Perfect agreement | 55 | 1.0000 |
| 1 | 0 | 0 | Within 1 case | 0 | |
| 2 | 1 | 1 | Within 2 cases | 0 | |
| 3+ | 0 | 0 | Within 3+ cases | 0 | |
| Prostate Cancer | | | | | |
| 0 | 54 | 48 | Perfect agreement | 48 | 0.1996 (-0.0907, 0.4899) |
| 1 | 1 | 6 | Within 1 case | 7 | |
| 2 | 0 | 1 | Within 2 cases | 0 | |
| 3+ | 0 | 0 | Within 3+ cases | 0 | |
| Skin Cancer | | | | | |
| 0 | 52 | 53 | Perfect agreement | 54 | 0.8518 (0.5436, 1.0000) |
| 1 | 2 | 1 | Within 1 case | 1 | |
| 2 | 1 | 1 | Within 2 cases | 0 | |
| 3+ | 0 | 0 | Within 3+ cases | 0 | |
| Lung Cancer | | | | | |
| 0 | 53 | 54 | Perfect agreement | 54 | 0.6584 (0.0349, 1.0000) |
| 1 | 2 | 1 | Within 1 case | 1 | |
| 2 | 0 | 0 | Within 2 cases | 0 | |
| 3+ | 0 | 0 | Within 3+ cases | 0 | |

**Table S2.** Participant demographics by full sample versus subset who did or did not completed qualitative interview.

| Demographic Variable | Full Sample (*N* = 56) | No Qualitative Interview (*N* = 26) | Completed Qualitative Interview (*N*=30) | *p*-Value |
|---|---|---|---|---|
| **Gender** | | | | |
| Male | 19 (34%) | 8 (30.8%) | 11 (36.7%) | 0.64 |
| Female | 37 (66%) | 18 (69.2%) | 19 (63.3%) | |
| **Age** | | | | |
| 21-24 | 3 (5.4%) | 3 (11.5%) | 0 (0%) | 0.07 |
| 25-34 | 12 (21.4%) | 5 (19.2%) | 7 (23.3%) | |
| 35-44 | 11 (19.6%) | 2 (7.7%) | 9 (30.0%) | |
| 45-54 | 10 (17.9%) | 4 (15.4%) | 6 (20.0%) | |
| 55-64 | 13 (23.2%) | 9 (34.6%) | 4 (13.3%) | |
| 65+ | 7 (12.5%) | 3 (11.5%) | 4 (13.3%) | |
| **Education** | | | | |
| < 9th grade | 10 (17.9%) | 3 (11.5%) | 7 (23.3%) | 0.96 |
| 9th-12th grade, no diploma | 10 (17.9%) | 5 (19.2%) | 5 (16.7%) | |
| High school degree/G.E.D. | 15 (26.8%) | 8 (30.8%) | 7 (23.3%) | |
| Some college, no degree | 4 (7.1%) | 2 (7.7%) | 2 (6.7%) | |
| Associate degree | 2 (3.6%) | 1 (3.8%) | 1 (3.3%) | |
| Bachelor's degree | 9 (16.1%) | 5 (19.2%) | 4 (13.3%) | |
| Graduate degree | 3 (5.5%) | 1 (3.8%) | 2 (6.7%) | |
| Post graduate degree (doctorate) | 3 (5.5%) | 1 (3.8%) | 2 (6.7%) | |
| **Income** | | | | |
| $25,000 or less | 27 (48.2%) | 14 (53.8%) | 13 (43.3%) | 0.57 |
| $25,001-$35,000 | 7 (12.5%) | 4 (15.4%) | 3 (10.0%) | |
| $35,001-$50,000 | 2 (3.6%) | 1 (3.8%) | 1 (3.3%) | |
| $50,001-$75,000 | 3 (5.4%) | 0 (0.0%) | 3 (10.0%) | |
| No answer | 17 (30.4%) | 7 (26.9%) | 10 (33.3%) | |
| **Country/US Territory of origin** | | | | |
| **Caribbean** | **34 (60.7%)** | **21 (80.8%)** | **13 (43.3%)** | 0.004* |
| Puerto Rico | 20 (35.7%) | 11 (42.3%) | 9 (30.0%) | |
| Dominican Republic | 14 (25%) | 10 (38.4%) | 4 (13.3%) | |
| **Central America** | **11 (19.6%)** | **2 (7.7%)** | **9 (30.0%)** | 0.036* |
| El Salvador | 7 (12.5%) | 1 (3.8%) | 6 (20.0%) | |
| Guatemala | 2 (3.6%) | 1 (3.8%) | 1 (3.3%) | |
| Honduras | 2 (3.6%) | 0 (0.0%) | 2 (6.6%) | |
| **South America** | **3 (5.4%)** | **1 (3.8%)** | **2 (6.7%)** | 1.00* |
| Ecuador | 1 (1.8%) | 0 (0.0%) | 1 (3.3%) | |
| Brazil | 1 (1.8%) | 1 (3.8%) | 0 (0.0%) | |
| Colombia | 1 (1.8%) | 0 (0.0%) | 1 (3.3%) | |
| Mexico | 8 (14.3%) | 2 (7.7%) | 6 (20.0%) | 0.26 |
| Spain | 0 (0.0%) | 0 (0.0%) | 0 (0.0%) | ----- |
| **Health literacy** | | | | |
| High likelihood of limited literacy | 19 (33.9%) | 8 (30.8%) | 11 (36.7%) | 0.61 |
| Possibility of limited literacy | 22 (39.3%) | 12 (46.2%) | 10 (33.3%) | |
| Almost always adequate literacy | 15 (26.8%) | 6 (23.1%) | 9 (30.0%) | |
| **Computer experience** | | | | |
| Never used one | 13 (23.2%) | 6 (23.1%) | 7 (23.3%) | 0.63 |
| Tried one a few times | 18 (32.1%) | 8 (30.8%) | 10 (33.3%) | |
| Use one regularly | 20 (35.7%) | 11 (42.3%) | 9 (30.0%) | |
| I'm an expert | 5 (8.9%) | 1 (3.8%) | 4 (13.3%) | |

* *p* -value represents comparisons between the block of countries versus not. E.g., Caribbean vs not Caribbean.

**Supplemental Material S3: Genetic Counselor Script.**

Hi _______, I'm ___________ a Genetic Counselor from ________________.

Today we're going to be talking about your family health history. I'm going to ask you some questions about your family and their health to try and get a sense of any health conditions that might run in your family. If you can't remember something or don't know the answer to any of the questions, don't worry, we'll work with what you do know. And don't worry about what you said or entered in the tool you used already, just focus on the questions we're going to talk about. Do you have any questions before we get started?

**Self**

How old are you _____? How is your health? Do you have any health problems?

*(If any health conditions are mentioned, the GC will follow up by asking when the condition was diagnosed, how it's being treated, along with any other relevant questions)*

Do you have any children______?

(If yes) How old is he/she? Does he/she have any health problems?

(If no) Is that by choice or because of infertility – or not being able to for medical reasons?

Do you have any adopted children______?

(If yes) How old is he/she? Does he/she have any health problems?

Do you have any grandchildren?

(If yes) How old? Does he/she have any health problems?

**Siblings**

Do you have any brothers or sisters? Do you and your siblings share the same mother and father? Do you have any half siblings?

Do any of your brothers or sisters have health problems?

*(If any health conditions are mentioned, the GC will follow up by asking when the condition was diagnosed, how it's being treated, along with any other relevant questions)*

Do your siblings have children?

(If yes) How old is he/she? Does he/she have any health problems?

(If no) Is that by choice or because of infertility?

**Parents**

Now let's talk about your parents – are they still living?

Does your Mom have any health problems?

*(If any health conditions are mentioned, the GC will follow up by asking when the condition was diagnosed, how it's being treated, along with any other relevant questions)*

Does your Mom have any brothers or sisters? Do your aunts/uncles have any health conditions?

*(If any health conditions are mentioned, the GC will follow up by asking when the condition was diagnosed, how it's being treated, along with any other relevant questions)*

Does your Mom's brother/sister have any children?

(If yes) How old is he/she? Does he/she have any health problems?

(If no) Is that by choice or because of infertility?

Are your Mom's parents still living? Do either of them have any health problems?

*(If any health conditions are mentioned, the GC will follow up by asking when the condition was diagnosed, how it's being treated, along with any other relevant questions)*

How is your Dad's health? Does he have any health problems?

*(If any health conditions are mentioned, the GC will follow up by asking when the condition was diagnosed, how it's being treated, along with any other relevant questions)*

Does he have any brothers or sisters? Do they have any health problems?

*(If any health conditions are mentioned, the GC will follow up by asking when the condition was diagnosed, how it's being treated, along with any other relevant questions)*

Does your Dad's brother/sister have any children?

(If yes) How old is he/she? Does he/she have any health problems?

(If no) Is that by choice or because of infertility?

Are your Dad's parents still living? Do either of them have any health problems?

*(If any health conditions are mentioned, the GC will follow up by asking when the condition was diagnosed, how it's being treated, along with any other relevant questions)*

**Conditions of Interest**

Thinking of everyone in the family, including those we have and have not already talked about, can you think of any family members that currently have any type of cancer or that had cancer in the past?

*(If any cancers are mentioned, the GC will follow up by asking about which relative, their current age, when the condition was diagnosed, how it's being treated, along with any other relevant questions)*

Can you think of any family members that have had any heart problems? Had a heart attack or have heart disease? Does anyone in the family have high cholesterol or high blood pressure, or take medication for these?

*(If any cardiovascular issues are mentioned, the GC will follow up by asking about which relative, their current age, when the condition was diagnosed or attack occurred, how it was/is being treated, along with any other relevant questions)*

What about diabetes? Is there anyone else in your family that you haven't mentioned that has diabetes?

*(If yes, the GC will follow up by asking about which relative, their current age, when the condition was diagnosed, how it's being treated, along with any other relevant questions)*

Other problems we'd like to know about are neurologic issues like stroke and Alzheimer's.

We'd also like to know about any addictions, including alcohol or drug addictions, and about any mental illness (like anxiety, depression, or schizophrenia).

Are there any other health conditions that any of your family members have had or currently have that you want to tell me about? Anything I haven't asked about and you think I should know?

What country did your family originally come from?

Is there a chance that your parents might be related by blood, like first or second cousins?

Thank you so much __________ . You will receive your gift card now. You also have the option of receiving a family tree, which would show all of the family members and health history information that we talked about today. Let *(research assistant)* know if you are interested in this and she will mail you a copy after your follow-up phone call.
